# Plasma protein alterations in the refractory anemia with excess blasts subtype 1 subgroup of myelodysplastic syndrome

**DOI:** 10.1186/1477-5956-10-31

**Published:** 2012-05-08

**Authors:** Pavel Májek, Zuzana Reicheltová, Jiří Suttnar, Jaroslav Čermák, Jan E Dyr

**Affiliations:** 1 Institute of Hematology and Blood Transfusion, U Nemocnice 1, 128 20 Prague 2, Czech Republic

**Keywords:** Myelodysplastic syndrome, RAEB-1, Plasma proteome, Refractory anemia

## Abstract

**Background:**

Refractory anemia with excess blasts subtype 1 (RAEB-1) is a subgroup of myelodysplastic syndrome. It represents a heterogeneous group of oncohematological bone marrow diseases, which occur particularly in elderly patients. The aim of this proteomic study was to search for plasma protein alterations in RAEB-1 patients.

**Results:**

A total of 24 plasma samples were depleted of fourteen high-abundant plasma proteins, analyzed with 2D SDS-PAGE, compared, and statistically processed with Progenesis SameSpots software. Proteins were identified by nanoLC-MS/MS. Retinol-binding protein 4 and leucine-rich alpha-2-glycoprotein were relatively quantified using mass spectrometry. 56 significantly differing spots were found; and in 52 spots 50 different proteins were successfully identified. Several plasma proteins that changed either in their level or modification have been described herein. The plasma level of retinol-binding protein 4 was decreased, while leucine-rich alpha-2-glycoprotein was modified in RAEB-1 patients. Changes in the inter-alpha-trypsin inhibitor heavy chain H4, altered protein fragmentation, or fragments modifications were observed.

**Conclusions:**

This study describes proteins, which change quantitatively or qualitatively in the plasma of RAEB-1 patients. It is the first report on qualitative changes in the leucine-rich alpha-2-glycoprotein in the RAEB-1 subgroup of myelodysplastic syndrome. Described changes in the composition or modification of inter-alpha-trypsin inhibitor heavy chain H4 fragments in RAEB-1 are in agreement with those changes observed in previous study of refractory cytopenia with multilineage dysplasia, and thus H4 fragments could be a marker specific for myelodysplastic syndrome.

## Background

Myelodysplastic syndrome (MDS) is a group of disorders affecting the blood and bone marrow, characterized by an increase in immature stem cells in the bone marrow, together with the presence of a higher count of abnormally developed cells. As a result, there is a significant decrease of one or more blood cell lines. Refractory anemia with excess blasts (RAEB) belongs to the group of MDS. According to World Health Organization (WHO) classification of MDS, two categories of RAEB are recognized based on the count of blasts in the bone marrow: RAEB-1 and RAEB-2 with 5-9% and 10-19% marrow blasts, respectively [1]. RAEB-1 is characterized by the presence of unilineage or multilineage dysplasia, and less than 5% blasts in the blood. Patients with RAEB-1 have a better clinical outcome than those with RAEB-2; nevertheless, a significant proportion of RAEB-1 patients may develop acute leukemia. Thus, specific markers that might characterize RAEB-l and may have a prognostic value, are currently under investigation.

Several genetic MDS studies have been published in recent years. For example, detailed differences in the expression of miRNAs between RAEB-1 and RAEB-2 have been observed in our institute [2]. On the contrary, very little is known about changes in plasma or serum proteins. There is a lack of proteomic data when searching for differences between RAEB-1 with RAEB-2, or when comparing RAEB-1 with other MDS categories. Zhong *et al.* found seven differentially expressed proteins (using Two-dimensional gel electrophoresis – 2-DE) in the sera of patients with RAEB in transformation, and suggested a possible role of dipeptidyl peptidase (DPP/CD26) in advanced MDS [3]. As alterations in plasma protein levels or in posttranslational modifications of proteins may play an important role in the disease’s mechanisms, the knowledge of such changes may be useful in disease diagnosis (especially when combined with modern methods using biosensors capable of fast and quantitative detection in plasma or blood [4-6]), treatment, and/or prognosis. Moreover, proteomic data could provide new additional information that is not possible to obtain when using genetic techniques alone [7].

The goal of our proteomic study was therefore to explore the plasma proteome of patients with RAEB-1, and to search for possible changes relative to healthy controls.

## Methods

Patients were diagnosed with RAEB-1 according to WHO classification criteria [1]. A total of 7 RAEB-1 patient plasma samples and 17 healthy controls were used in the proteomic analysis. RAEB-1 patient age ranged from 58 to 71 years, and there were 4 males (57%). Healthy control donor age ranged from 20 to 35, and there were 9 males (53%). All tested individuals agreed to participate in this study and gave informed consent. All samples were obtained and analyzed in accordance with the Ethical Committee regulations of the Institute of Hematology and Blood Transfusion.

Blood (10 mL) was collected by venipuncture into tubes coated with EDTA, centrifuged (5 min, 4000xg), and plasma aliquots were then transferred to polypropylene Eppendorf tubes and stored at −70°C until used. There was no sample pooling (2D electrophoresis, western blotting and MS/MS based relative label-free protein quantification) used in this study – non-pooled individual samples of patients and donors were used only. Thawed plasma samples were centrifuged (5 min, 12000xg), and a MARS Hu-14 4.6 x 100 mm column (Agilent, Santa Clara, CA, USA) was used to remove fourteen high-abundant proteins (albumin, IgG, antitrypsin, IgA, transferrin, haptoglobin, fibrinogen, alpha2-macroglobulin, alpha1-acid glycoprotein, IgM, apolipoprotein AI, apolipoprotein AII, complement C3, and transthyretin), according to manufacturer instructions. 5 K MWCO Spin Concentrators (Agilent) were used to desalinate and concentrate the samples (3000xg, 20°C). MilliQ water (4 mL) was added to the concentrated samples, and the desalinating-concentrating step was repeated three times. Finally, desalinated and concentrated samples were vacuum dried, rapidly frozen, and stored at −70°C.

2D electrophoresis, followed by image analysis, protein in-gel digestion and mass spectrometry identification were performed as described in previous publications [7-9]. Briefly, isoelectric focusing (IPG strips pI 4–7, 7.7 cm), followed by SDS-PAGE (8 x 10 cm, 10% gel, 3.75% stacking gel, 5°C, 30 mA/gel), was used in the first and second dimension, respectively. Protein alkylation was performed during equilibration of IPG strips before SDS-PAGE. Gels were stained using colloidal Coomassie blue, scanned, and processed with Progenesis SameSpots software (Nonlinear Dynamics, Newcastle upon Tyne, UK), which computed the fold change (the fold difference of logarithms of normalized spot volumes between the patient and control groups; the average values of normalized spot volume logarithms of a single spot in all samples within a group are compared) and p-values of all spots using one-way ANOVA analysis. All patient and control samples were used for 2D electrophoresis. No technical replicates were used. Principal Component Analysis (PCA) was performed to assess whether grouping of patients and healthy controls based on proteomic methods reflects their stratification using classical clinical diagnosis. PCA was performed making use of the same software, focusing only on the spots of statistical significance (based on 2D SDS-PAGE) employed for protein identification. Differing spots were excised from the gel, and the proteins were in-gel digested with trypsin. MS/MS mass spectrometry (HCT ultra ion-trap mass spectrometer with nanoelectrospray ionization; Bruker Daltonics, Bremen, Germany) coupled to a nanoLC system (UltiMate 3000; Dionex, Sunnyvale, CA, USA) was used to perform MS analysis. MASCOT (Matrix Science, London, UK) was used for database searching (Swiss-Prot release 2010_12). To eliminate peptide carry-over between HPLC separations, cleaning runs were performed before and after each sample run. Two unique peptides (with higher scores than the minimum, p < 0.05) were necessary to successfully identify a protein.

To compare the plasma levels of both leucine-rich alpha-2-glycoprotein (LRAG) and retinol-binding protein 4 (RBP4) in patient and control samples, relative label-free protein quantification was used on the basis of Selected Ion Monitoring (SIM). At first, plasma proteins were digested using modified protocols of the acetonitrile precipitation of plasma proteins and trypsin digestion, as described by Kay *et al.*[10]. Briefly, 100 μl of water and 225 μl of acetonitrile were added to 50 μl of plasma, and sonicated twice for 10 min with vortexing between sonications. After centrifugation (10 min, 12000xg) 300 μl of supernatant were transferred into a new Eppendorf tube and dried completely. Pellets were then resuspended in 40 μl of 0.1 M NH_4_HCO_3_, 5 μl of 0.1 M DTT were added, and the samples were incubated for 1 hr at 60°C. After the samples cooled, 5 μl of 0.1 M iodoacetamide were added, and the samples were incubated at room temperature in the dark for 30 min. Trypsin digestion was induced by adding 4 μl of trypsin (200 μg/mL) in 50 mM acetic acid and digested at 37°C overnight. The reaction was stopped with the addition of 6.5 μl of 1% formic acid. Samples were centrifuged (10 min, 12000xg), filtered, and 20 μl of each sample were loaded for LC-MS/MS analysis. SIM for LRAG was performed by monitoring two peptides: ENQLEVLEVSWLHGLK with precursor ion 947.5 m/z (charge 2+) and product ion 1181.8 m/z (y10) as described by Kay *et al.*[11]; and TLDLGENQLETLPPDLLR with precursor ion 1019.1 m/z (2+) and product ion 710.4 m/z (y6). SIM for RBP4 was performed by monitoring FSGTWYAMAK with precursor ion 589.3 m/z (2+) and product ion 785.3 m/z (y6), and YWGVASFLQK with precursor ion 599.9 m/z (2+) and two product ions – 693.4 m/z (y6) and 849.5 m/z (y8). Peptides, precursor, and product ions were chosen on the basis of MS/MS analysis (previous protein identification). The nanoLC-MS/MS system was the same as described above, but using a 60 min 0-70% acetonitrile non-linear gradient; with a precursor selection window width of 2 Da. Extracted ion chromatograms of the product ions were generated with an ion m/z width of ± 0.5 Da; and peak areas were calculated after automatic integration using DataAnalysis software (version 4.0; Bruker). Each sample was measured twice. All sample results were validated according to MS/MS spectra and retention times [12]. Blank runs were performed before and after each sample run to eliminate peptide carry-over.

Western blotting was performed as described previously [7]. Briefly, proteins were transferred from a gel (corresponding to the second dimension as used in 2D SDS-PAGE) to a PVDF membrane (10 V constant voltage for 1 hr); the membrane was then incubated with a blocking buffer (3% BSA in PBS) at 4°C overnight, rinsed, and incubated with a primary antibody anti-ITIH4 (Abnova, Taipei, Taiwan); 1:2000 dilution, at 30°C for 45 min. The membrane was then incubated with the secondary antibody (rabbit anti-mouse IgG antibody conjugated with peroxidase; Sigma-Aldrich, Prague, Czech Republic), 1:60000 dilution, at 30°C for 45 min. After rinsing, a chemiluminescent substrate (SuperSignal West Pico; Thermo Scientific, Waltham, MA, USA) was added to the membrane for 5 min; and an appropriate film exposition (Amersham Hyperfilm ECL; GE Life Sciences, Piscataway, NJ, USA) was performed.

## Results

**Figure 1:**
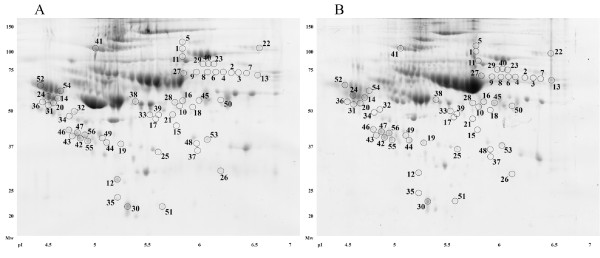
**Positions of significantly differing spots on a 2D gel.** Positions of all spots that were found to differ significantly when RAEB-1 patients were compared to healthy controls. The 2D gels of a patient sample ( **A**) and a healthy control ( **B**) were used as illustrative gels to display spot positions.

**Figure 2:**
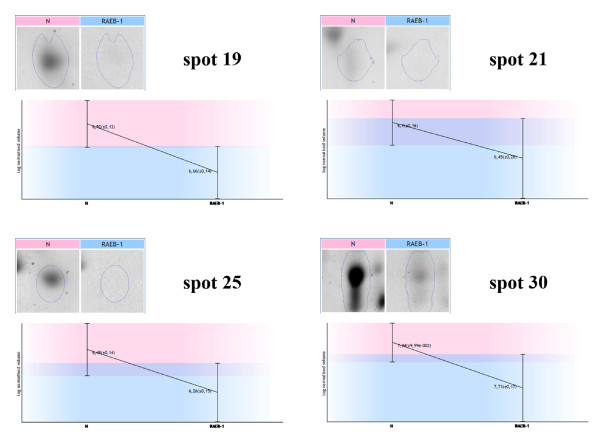
**An example of four detected spots.** Expression profiles with the logarithms of spot normalized volumes and their standard deviations for RAEB-1 patient and healthy control groups are presented for four (19, 21, 25, and 30) spots.

Comparing the RAEB-1 group (n = 7) with the control group (n = 17), 56 spots that differed significantly (p < 0.05, ANOVA) in their normalized volumes were found (Figure 1). Proteins in 52 spots were successfully identified using MS/MS, and corresponded to 50 unique proteins. All spots and identified proteins are summarized in the list of spots; including ANOVA p-value, fold change, protein identification with the number of identified peptides (unique peptides above the threshold score), protein accession number (Swiss-Prot), and sequence coverage (See additional file 1: Table S1). An example of four spots presented in Table S1 is shown in Figure 2.

Fragmentation of the inter-alpha-trypsin inhibitor heavy chain H4 (ITIH4) was observed in several spots (17, 19, 25, 42, 44, 47, 55, and 56). In spite of the presence of co-identified proteins in all spots except spot 19, a trend of decreased normalized volumes of each spot in RAEB-1 was observed, when compared with the control group. Comparing the sequences of ITIH4 identified peptides (using MS/MS) in all spots with the observed fragmentation, these peptides corresponded to the 35 kDa ITIH4 processed protein. In addition, the molecular weights of the spots with the observed ITIH4 fragmentation, as estimated using 2D SDS-PAGE, corresponded to that of 35 kDa ITIH4. Uncleaved ITIH4 was identified in spot 41.

**Figure 3:**
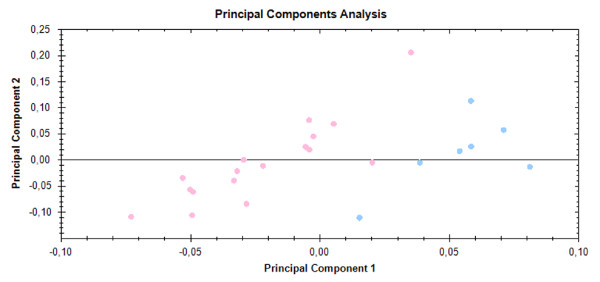
**Principal Component Analysis.** PCA was performed to assess whether grouping of patient and healthy controls based on proteomic methods (2D SDS-PAGE) reflects their stratification using classical clinical diagnosis. Analysis was based on spots that significantly differed according to the mentioned statistical criteria (p < 0.05, ANOVA). PCA showed the separation of all samples into two aggregates that corresponded to the RAEB-1 (blue dots) and healthy control group (pink dots).

Principal Component Analysis showed an obvious separation of samples into two aggregates, corresponding to the RAEB-1 and control groups (Figure 3).

Relative label-free quantification of LRAG and RBP4 was performed using all seven RAEB-1 samples (the same samples as used in 2D SDS-PAGE) and compared to seven sex-matched control samples from healthy donors. Each sample was measured twice, and average values of peak areas of peptides in patient and control groups were compared. Quantification of LRAG showed different fold change values for its peptides. The average peak area value was more than two-fold decreased in the MDS group (2.24-fold) when compared with the control group for 1019.1/710.4, while there was no obvious difference for 947.5/1181.8 (1.08-fold). Quantification of RBP4 showed a decrease in the both measured peptides in the MDS group. There were two product ions (693.4 m/z – y6, and 849.5 m/z – y8) measured for YWGVASFLQK (599.9 m/z) to establish which would be suitable to use for SIM or possible Selected Reaction Monitoring (SRM). Both transitions were found to be suitable and stable with similar results. For both there was more than a three-fold decrease in the MDS group when compared with the control group (3.21-fold and 3.29-fold for 599.9/693.4 and 599.9/849.5, respectively). For the second RBP4 peptide (589.3/785.3), more than a two-fold decrease (2.33-fold) was observed in the patient group. The results are summarized in Table 1.

**Table 1:** Relative label-free quantification of proteins

**Protein**	**Peptide sequence**	**Precursor ion [m/z]**	**Precursor charge**	**Product ion [m/z]**	**Product ion**	**fold (N/MDS)**
LRAG	TLDLGENQLETLPPDLLR	1019.1	2+	710.4	y6	2.24
LRAG	ENQLEVLEVSWLHGLK	947.5	2+	1181.8	y10	1.08
RBP4	FSGTWYAMAK	589.3	2+	785.3	y6	2.33
RBP4	YWGVASFLQK	599.9	2+	693.4	y6	3.21
RBP4	YWGVASFLQK	599.9	2+	849.5	y8	3.29

LRAG (leucine-rich alpha-2-glycoprotein), RBP4 (retinol-binding protein 4).

The plasma level of uncleaved ITIH4 was assessed by western blot analysis. No difference in ITIH4 expression between the patient group (n = 7) and the control group (n = 7) was observed.

## Discussion

Changes in the plasma proteome of RAEB-1 patients are presented in this paper. Further, two proteins, leucine-rich alpha-2-glycoprotein and retinol-binding protein 4 that were uniquely identified within their spots and could be of interest as they have not been associated with MDS before, were quantified relatively using SIM to validate the data obtained by 2D electrophoresis. The criteria for selection of the two proteins for further validation were their unique identification within a spot, fold, and p-values. As the presence of other proteins in a spot may influence the spot normalized volume and consequently the fold and p-value, we did not consider non-uniquely identified proteins for further validation (relative quantification).

When summarizing our data, the main factor that seemed to influence the results and participate the most in plasma proteome changes was inflammation. The majority of the identified proteins that changed were proteins of acute phase reaction and those involved in inflammation response: C-reactive protein, prothrombin, complement proteins, alpha-1-antichymotrypsin, albumin, etc. These results are in agreement with the previously shown involvement of inflammation in MDS [13-15]. Inflammation and expression of the inflammation response proteins are closely related to apoptosis and to the production of inflammatory cytokines, a unifying factor of different MDS subgroups.

In this study, alterations of the inter-alpha-trypsin inhibitor heavy chain H4 (ITIH4) fragments were observed. ITIH4 is a protein involved in acute phase reaction and is considered to be a possible marker of cancer [16]. Its fragments have been found to be potentially associated with MDS [17]; with differences in the fragmentation of ITIH4 having been shown in literature [16,18] and related to several malignant diseases [18-21]. Moreover, changes in the composition or modifications in ITIH4 fragments were observed in our previous proteomic study describing plasma proteome changes in another MDS subgroup – refractory cytopenia with multilineage dysplasia (RCMD) [22]. In RAEB-1, ITIH4 peptide sequences (according to MS/MS) and spot molecular weights (estimated by 2-DE) corresponded to the 35 kDa processed ITIH4 protein (as processed by kallikrein) in all spots except spot 41, which corresponded to uncleaved ITIH4. Normalized volumes of the spots containing processed 35 kDa ITIH4 fragments were decreased. The same results were found in RCMD patients [22]. Uncleaved ITIH4 plasma levels estimated with western blotting in both RAEB-1 and RCMD patients, and also in control groups were the same. Therefore, the increase of normalized volumes of spots containing uncleaved ITIH4 (both in RAEB-1 and RCMD) was most likely caused by co-identification. As the results for ITIH4 fragments in the RAEB-1 group are the same as in RCMD patients (spot positions within 2D maps, fold changes, estimated fragment molecular weights, and western blot analysis results) we suppose that the changes in the composition or modifications of ITIH4 fragments may be specific for all myelodysplastic syndromes and not only for its subgroups. Nevertheless, comparisons of the plasma proteomes of other MDS subgroups are necessary to draw further conclusions. The results obtained by 2-DE concerning ITIH4 fragments may be explained by a progressive fragmentation of ITIH4 fragments in the patient group producing smaller fragments (which could not be detected by 2-DE as used in our study); as well as also the possibility of posttranslational modifications. Nevertheless, in light of the observations from RAEB-1, RCMD, and previous literature [17] it seems that ITIH4 alterations could be a marker specific for myelodysplastic syndrome.

Leucine-rich alpha-2-glycoprotein (LRAG) was identified in two spots (31 and 36), with normalized volumes increased in RAEB-1 by 1.7- and 1.5-fold, respectively. LRAG, isolated for the first time in 1977 by Haupt and Baudner [23], contains 8 repeating sequences (each consisting of 24 amino acids) termed leucine-rich repeat (LRR). Since the first isolation and identification of this protein, many proteins have been identified to contain LRR sequences, probably playing a significant role in protein-protein interactions. The physiological function of LRAG remains unknown, although some clues have been observed (LRAG was proposed to possibly be involved in the innate immune response) [24]. It has been also suggested that LRAG may be a marker of granulocytic differentiation; moreover, it was reported that its expression was up-regulated during neutrophil differentiation [25]. Altered serum expression (up-regulation) of LRAG was observed in several diseases, for example in lung cancer [26] and pancreas cancer [27]. It has been reported that the expression of LRAG in human hepatoma cells was induced by IL-6, IL-1β, and TNFα; and that the LRAG expression was enhanced by the induction of acute inflammation in mice [24]. As the LRAG alteration in RAEB-1 using 2D electrophoresis was observed, a relative quantification using mass spectrometry was further performed to establish whether the LRAG change was based on the altered expression only, or if it was affected by posttranslational modification. The need for confirmation was supported by the fact that LRAG was identified in two spots with the same molecular weight but different pI, indicating the presence of posttranslational modification(s) (for example, LRAG glycosylation has been observed [28-30]; however, this was not examined in this study). The relative quantification of two LRAG peptides showed completely different results when compared to 2D electrophoresis results: the first peptide (SIM of 947.5/1181.8) was not altered at all, while the second peptide was actually decreased more than twice in the MDS group. This difference proved the presence of posttranslational modification(s) of LRAG in MDS (no alterations in different peptides of the same protein should be present in case of level change only), and it is likely that the LRAG plasma level is not changed. However, the possibility of LRAG plasma level changes has not been excluded. In our previous study of RCMD, LRAG was identified only in one spot and as the only protein in the spot [22]. Even though the number of plasma samples was substantially higher than in this RAEB-1 study (22 RCMD patients and 24 controls), the p-value was higher (p = 0.043) and the fold value was lower (1.3). Therefore, LRAG or its modification(s) could be a RAEB-1 specific marker.

Retinol-binding protein 4 (RBP4) was identified in spot 30, with its normalized volume decreased in RAEB-1 by 1.7-fold. RBP4 is a specific carrier protein for retinol, which is delivered from the liver to the peripheral tissues. The binding of retinol to RBP4 facilitates the transfer of retinol, which is insoluble in aqueous solutions [31]. It has been shown that RBP4 is associated with variables related to insulin resistance and diabetic complications [32]; an increased level of plasma RBP4 increases insulin resistance by inhibiting insulin signaling [33]. RBP4 was also considered to be involved in inflammation, but no correlation was found between RBP4 and C-reactive protein in a study of RBP4 in type 2 diabetes patients [32]. In a more recent study, the RBP4 serum level was significantly reduced in critically ill patients independently of sepsis (as compared with the healthy control group), and their RBP4 level was closely correlated with liver function [34]. Finally, acute phase proteins were inversely correlated with RBP4 in sepsis patients. As in the case of leucine-rich alpha-2-glycoprotein, relative quantification using mass spectrometry (SIM) was performed to differentiate between the RBP4 plasma level change and possible posttranslational modification. Two RBP4 peptides were monitored and two product ions (y6 and y8) of the first peptide were measured (599.9/693.4 and 599.9/849.5) to find a proper transition. Both y6 and y8 product ions were found to be suitable with a stable response and duplicate results: a decrease in the MDS group of 3.21-fold and 3.29-fold, which averages 3.25-fold for the first peptide. The second RBP4 peptide was decreased as well with a fold change of 2.33. These results were in compliance with those observed by 2D electrophoresis, suggesting that the RBP4 plasma level is markedly decreased in RAEB-1 patients. In our previous study of RCMD, RBP4 was co-identified in one spot only; moreover, its normalized volume was increased in RCMD in contrast to RAEB-1 results. Therefore, it seems that RBP4 could be a RAEB-1 specific marker. It will nevertheless, be necessary to compare our results to other MDS subgroups to determine whether the decrease of the RBP4 plasma level is related to MDS pathophysiology specific for RAEB-1, or if it is a result of inflammation, as the RBP4 is a negative acute phase protein and its level is decreased during the inflammatory response.

An additional issue that must be discussed is the disparity of age ranges between the patient and healthy control groups. The optimal control group should of course match for age as well as for sex. As MDS usually occurs in elderly patients, there should be a control group of a similar age range. However, this matching may contribute to other limitations – the primary difficulty is to find healthy individuals of older age, who do not suffer from other complicating diseases. These variables may significantly affect our obtained results. Moreover, MDS is not exclusively limited to elderly patients only. The problem of collecting and matching of the control groups including our methodology and further justification was discussed in detail in our previous MDS study [22].

## Conclusions

In conclusion, this study represents the first report on the qualitative alteration in leucine-rich alpha-2-glycoprotein, and the change in retinol-binding protein 4 in the plasma of MDS patients with RAEB-1. The changes in both proteins seem to be specific for RAEB-1, when compared to another subgroup of myelodysplastic syndrome – refractory cytopenia with multilineage dysplasia. We may speculate that this alteration of LRAG may reflect the impairment of granulocyte differentiation, which is linked to disease progression. Since the level of proapoptotic cytokines affects the level of both proteins, we may also speculate that their decrease might reflect a decreased rate of apoptosis, also connected with a progressive disease. Furthermore, changes in the composition or modifications of the inter-alpha-trypsin inhibitor heavy chain H4 fragments were observed. These changes were in agreement with those observed in the refractory cytopenia with multilineage dysplasia subgroup of MDS. Proteomic studies of other MDS subgroups will help to find proteins that are universally changed in MDS when compared with the healthy population, or specifically altered in different subgroups of MDS.

## Competing interests

The author(s) declare that they have no competing interests.

## Authors' contributions

PM and ZR designed and performed research, analyzed data, and wrote the manuscript. JC diagnosed the patients and collected the samples. JS, JC, and JED designed research and wrote the manuscript. All authors have read and approved the final manuscript.

## Supplementary Material

Addtional file 1 List of spots that differ significantly when RAEB-1 patients and healthy controls were compared. Click here for file
