# Supplementary material for: Plasma protein alterations in the refractory anemia with excess blasts subtype 1 subgroup of myelodysplastic syndrome
Source: Proteome Sci. 2012 May 8;10:31. doi: 10.1186/1477-5956-10-31 (PMC3470985; doi:10.1186/1477-5956-10-31)
Supplement: Addtional file 1 — List of spots that differ significantly when RAEB-1 patients and healthy controls were compared. [file 1477-5956-10-31-S1.pdf]

Table S1. List of spots that differ significantly when RAEB-1 patients and healthy controls were compared.

spot	p	fold	protein	peptides	AC	SC (%)
1	0.0009	-6.6	Serum albumin	13	P02768	33
			Plasma protease C1 inhibitor	4	P05155	12
2	0.00031	-4.0	Serum albumin	14	P02768	38
			Fibrinogen alpha chain	2	P02671	3
3	0.00048	-3.8	Serum albumin	13	P02768	37
			Hemopexin	4	P02790	18
4	0.003	-3.7	Serum albumin	13	P02768	32
			Protein Z-dependent protease inhibitor	2	Q9UK55	5
5	0.00037	-3.5	Serum albumin	12	P02768	35
			Plasma protease C1 inhibitor	2	P05155	8
			Inter-alpha-trypsin inhibitor heavy chain H2	2	P19823	3
			Complement component C6	3	P13671	5
6	0.007	-3.1	Serum albumin	15	P02768	44
			Protein Z-dependent protease inhibitor	2	Q9UK55	16
			Histidine-rich glycoprotein	2	P04196	4
7	0.002	-3.0	Serum albumin	13	P02768	35
			Fibrinogen alpha chain	2	P02671	5
			Hemopexin	4	P02790	20
			Alpha-1-antichymotrypsin	2	P01011	8
			Complement component C8 beta chain	2	P07358	7
8	0.017	-2.8	Serum albumin	14	P02768	43
			Protein Z-dependent protease inhibitor	2	Q9UK55	9
			Hemopexin	2	P02790	20
9	0.009	-2.7	Serum albumin	20	P02768	55
			Protein Z-dependent protease inhibitor	2	Q9UK55	16
			Histidine-rich glycoprotein	2	P04196	4
10	0.006	-2.5	Serum albumin	6	P02768	13
			Beta-2-glycoprotein 1	3	P02749	24
11	0.012	-2.5	Serum albumin	16	P02768	42
			Gelsolin	6	P06396	15
			Plasma protease C1 inhibitor	3	P05155	11
			Coagulation factor XIII B chain	2	P05160	10
12	0.002	2.4	Apolipoprotein A-I	3	P02647	32
			C-reactive protein	4	P02741	20
			Ig kappa chain C region	2	P01834	32
13	0.006	-2.3	Serum albumin	14	P02768	30
			Complement C4-A; (B)	5	P0C0L4;P0C0L5	5
			Complement component C8 beta chain	2	P07358	7
			Alpha-1-antichymotrypsin	2	P01011	14
			Fibrinogen alpha chain	2	P02671	8
			Hemopexin	3	P02790	18
14	0.00001	-2.2	Alpha-1-antichymotrypsin	4	P01011	21
			Kininogen-1	6	P01042	14
			Antithrombin-III	2	P01008	5
15	0.01	-2.1	Serum albumin	9	P02768	17
16	0.007	-2.0	Beta-2-glycoprotein 1	6	P02749	25
			Serum albumin	3	P02768	11
			Complement factor I	2	P05156	6
17	0.001	-2.0	Serum albumin	3	P02768	14
			Inter-alpha-trypsin inhibitor heavy chain H4	3	Q14624	7
18	0.0008	-1.9	Serum albumin	4	P02768	13
			Pigment epithelium-derived factor	3	P36955	12
			Hemopexin	2	P02790	12
19	0.002	-1.9	Inter-alpha-trypsin inhibitor heavy chain H4	5	Q14624	8
20	0.001	-1.8	Kininogen-1	2	P01042	4
21	0.022	-1.8	Serum albumin	8	P02768	24
			Pigment epithelium-derived factor	4	P36955	19
22	0.015	-1.8	Plasminogen	8	P00747	29
			Complement C5	3	P01031	2
			Complement C4-A; (B)	4	P0C0L4;P0C0L5	4
			Serum albumin	2	P02768	12
			Complement factor B	2	P00751	5
23	0.003	1.8	Prothrombin	9	P00734	21
			Ig mu chain C region	5	P01871	21
			Histidine-rich glycoprotein	3	P04196	11
24	0.0006	-1.7	Alpha-1-antichymotrypsin	2	P01011	11
			Antithrombin-III	3	P01008	12
			Serum albumin	2	P02768	4
25	0.01	-1.7	Inter-alpha-trypsin inhibitor heavy chain H4	5	Q14624	11
			Serum amyloid P-component	2	P02743	9
26	0.005	1.7	Ig kappa chain C region	3	P01834	80
			Ig lambda-2 chain C regions (3,6,7)	2	P0CG05	32
			Ig kappa chain V-III region SIE (Ti, WOL, GOL)	2	P01620	31
27	0.01	-1.7	Serum albumin	23	P02768	67
28	0.012	-1.7	Serum albumin	9	P02768	20
			Pigment epithelium-derived factor	6	P36955	22
			Complement factor I	3	P05156	14
29	0.018	1.7	Ig mu chain C region	6	P01871	26
			Serum albumin	3	P02768	11
			Histidine-rich glycoprotein	3	P04196	7
			Prothrombin	5	P00734	26
			Alpha-1B-glycoprotein	2	P04217	10
30	0.001	-1.7	Retinol-binding protein 4	3	P02753	62
31	0.008	1.7	Leucine-rich alpha-2-glycoprotein	8	P02750	42
32	0.044	1.7	unidentified			
33	0.017	-1.6	Serum albumin	9	P02768	28
34	0.032	1.6	Haptoglobin	4	P00738	14
			Hemopexin	2	P02790	12
			Zinc-alpha-2-glycoprotein	2	P25311	19
35	0.002	1.6	C-reactive protein	2	P02741	16
36	0.035	1.5	Leucine-rich alpha-2-glycoprotein	4	P02750	15
37	0.042	-1.5	unidentified			
38	0.003	-1.5	Antithrombin-III	7	P01008	43
			Angiotensinogen	3	P01019	11
			Monocyte differentiation antigen CD14	5	P08571	23
			Vitamin D-binding protein	3	P02774	18
			Histidine-rich glycoprotein	3	P04196	12
			Hemopexin	2	P02790	10
			Fibrinogen gamma chain	3	P02679	25
39	0.02	-1.5	Serum albumin	3	P02768	14
40	0.023	1.5	Ig mu chain C region	2	P01871	13
			Prothrombin	3	P00734	25
			Inter-alpha-trypsin inhibitor heavy chain H1	2	P19827	6
			Alpha-1B-glycoprotein	2	P04217	8
41	0.035	1.4	Inter-alpha-trypsin inhibitor heavy chain H4	15	Q14624	26
			Alpha-1-antitrypsin	5	P01009	22
			Angiotensinogen	4	P01019	14
			Prothrombin	3	P00734	24
			Thyroxine-binding globulin	2	P05543	4
			Ceruloplasmin	2	P00450	2
42	0.006	-1.4	Clusterin	2	P10909	12
			Inter-alpha-trypsin inhibitor heavy chain H4	2	Q14624	3
43	0.01	-1.4	unidentified			
44	0.022	-1.4	Inter-alpha-trypsin inhibitor heavy chain H4	5	Q14624	11
			Protein AMBP	3	P02760	20
45	0.016	-1.4	Hemopexin	8	P02790	28
			Alpha-1-antichymotrypsin	2	P01011	8
			Beta-2-glycoprotein 1	3	P02749	24
			Serum albumin	2	P02768	10
46	0.015	-1.4	Clusterin	2	P10909	11
47	0.005	-1.4	Clusterin	2	P10909	11
			Inter-alpha-trypsin inhibitor heavy chain H4	2	Q14624	3
48	0.019	1.4	unidentified			
49	0.044	-1.4	Complement factor I	2	P05156	3
50	0.017	-1.4	Hemopexin	5	P02790	25
			Alpha-1-antichymotrypsin	2	P01011	8
			Serum albumin	2	P02768	7
			Kallistatin	2	P29622	13
			Beta-2-glycoprotein 1	2	P02749	20
51	0.018	-1.3	Tetranectin	3	P05452	13
52	0.007	1.3	Alpha-1-antichymotrypsin	14	P01011	46
			Corticosteroid-binding globulin	2	P08185	11
53	0.02	1.3	Alpha-1-antichymotrypsin	2	P01011	14
54	0.032	-1.3	Alpha-1-antichymotrypsin	7	P01011	45
			Angiotensinogen	4	P01019	12
			Kininogen-1	8	P01042	19
			Beta-Ala-His dipeptidase	2	Q96KN2	19
55	0.029	-1.2	Clusterin	2	P10909	15
			Inter-alpha-trypsin inhibitor heavy chain H4	2	Q14624	5
56	0.041	-1.2	Clusterin	2	P10909	16
			Inter-alpha-trypsin inhibitor heavy chain H4	2	Q14624	6
			Complement C3	2	P01024	6

p - ANOVA p-value; **fold** - fold difference (multiplication) when RAEB-1 patients and healthy controls compared (+fold when the normalized volumes increased in RAEB-1); **protein** - protein identification; **peptides** - number of unique peptides fulfilling a minimal Mascot score; **AC** - accession number (SWISS-PROT); **SC** - protein sequence coverage
